# Sociodemographic characteristics and quality of life between persons with addiction disorders and their caregivers

**DOI:** 10.1192/j.eurpsy.2025.837

**Published:** 2025-08-26

**Authors:** O. B. Sbutega Filipovic, D. Raketic, R. Peric

**Affiliations:** 1 Day hospital for chemical and non-chemical addictions; 2 Special Hospital on Addictions, Belgrade, Serbia

## Abstract

**Introduction:**

Disorders caused by alcohol and opiate addiction lead to physical, mental and socioeconomic deterioration not only of the patient, but also of their families. Family caregivers are persons who provide unpaid care to other family members who need supervision or help in case of illness or disability, as well as to persons with special needs (1). Studies have shown that the illness of one family member affects the quality of life of other family members, especially the caregiver of the patient (2). Previous studies indicate that the support of family members is of great importance and influence on the initiation of addiction treatment, compliance and participation in it, but also on the outcome itself, i.e. the success of the treatment (3).

**Objectives:**

Primary aims of this study included sociodemographic characteristics and analysis of the quality of life between persons with addiction disorders and their caregivers.

**Methods:**

The study included 136 patients who were being treated at the Special Hospital for Addiction Diseases in Belgrade, for the treatment of addiction to psychoactive substances (opiates and opioids or alcohol), and 136 of their caregivers. Data on respondents were collected in the period from April to October 2014. During this research, a cross-sectional study was conducted. As measuring instruments in this research, in addition to the general questionnaire, specific questionnaire was used for assessment of quality of life (*36 item Short-Form Health Survey – SF-36* (4,5).

**Results:**

Sociodemographic characteristics indicate that there are more male addicts, as well as female caregivers (p< 0.001). Psychoactive substance addicts belong to the age group ≤39 years, compared to caregivers who belong to the group older than 50 years (p<0.001). The marital status of the respondents showed that the largest percentage of patients were without a partner, while the same number of guardians were married (p<0.001). The socio-economic status of the respondents showed a statistically significant difference in relation to the socioeconomic conditions of the patients and their caregivers. SF-36 domain scores in caregivers were similar to those in with addictive disorder, with the exception that 2 domains were significantly lower, related to physical functioning (p < 0.001) and bodily pain (p = 0.003). A greater number of patients report a better state of health compared to last year compared to caregivers who consider their health to be the same or worse in the mentioned period (p<0.001).

**Conclusions:**

Assessment of the quality of life of persons addicted to psychoactive substances, as well as their caregivers, is an important aspect of the therapeutic protocol in order to provide the necessary help and more successful treatment.

**Disclosure of Interest:**

None Declared

